# Neurobehavioral and Ultrastructural Changes Induced by Phytosynthesized Silver-Nanoparticle Toxicity in an In Vivo Rat Model

**DOI:** 10.3390/nano12010058

**Published:** 2021-12-26

**Authors:** Razvan Vlad Opris, Vlad Toma, Alina Mihaela Baciu, Remus Moldovan, Bogdan Dume, Alexandra Berghian-Sevastre, Bianca Moldovan, Simona Clichici, Luminita David, Gabriela Adriana Filip, Adrian Florea

**Affiliations:** 1 Department of Physiology, “Iuliu Hatieganu” University of Medicine & Pharmacy, 1-3 Clinicilor Street, 400006 Cluj-Napoca, Romania; opris.razvan93@yahoo.com (R.V.O.); alinabacium@gmail.com (A.M.B.); remus_ri@yahoo.com (R.M.); alexandra_berghian@yahoo.com (A.B.-S.); simonaclichici@yahoo.com (S.C.); 2 Department of Biochemistry & Experimental Biology, Institute of Biological Research, 48 Republicii Street, Branch of NIRDBS Bucharest, 400015 Cluj-Napoca, Romania; vlad.al.toma@gmail.com; 3 National Institute for Research and Development of Isotopic and Molecular Technologies, 67-103 Donath Street, 400293 Cluj-Napoca, Romania; 4 Department of Molecular Biology and Biotechnologies, “Babeș-Bolyai” University, 4-6 Clinicilor Street, 400006 Cluj-Napoca, Romania; 5 Department of Chemistry, Faculty of Chemistry & Chemical Engineering, “Babeș-Bolyai” University, 11 Arany Janos Street, 400028 Cluj-Napoca, Romania; bobihas@gmail.com (B.D.); bianca@chem.ubbcluj.ro (B.M.); luminita.david@ubbcluj.ro (L.D.); 6 Department of Cell & Molecular Biology, “Iuliu Hatieganu” University of Medicine & Pharmacy, 6 Louis Pasteur Street, 400349 Cluj-Napoca, Romania; adrian_a_florea@yahoo.com

**Keywords:** electron microscopy, silver nanoparticles, rat brain, green synthesis, neurotoxicity

## Abstract

(1) Background: The study aimed to assess neurobehavioral, ultrastructural, and biochemical changes induced by silver nanoparticles synthesized with *Cornus mas* L. extract (AgNPs-CM) in rat brains. (2) Methods: The study included 36 male adult rats divided into three groups. Over a period of 45 days, AgNPs-CM (0.8 and 1.5 mg/kg b.w.) were administered daily by gavage to two of the groups, while the control group received the vehicle used for AgNP. After treatment, OFT and EPM tests were conducted in order to assess neurobehavioral changes. Six of the animals from each group were sacrificed immediately after completion of treatment, while the remaining six were allowed to recuperate for an additional 15 days. Transmission electron microscopy (TEM), GFAP immunohistochemistry, and evaluation of TNFα, IL-6, MDA, and CAT activity were performed on the frontal cortex and hippocampus. (3) Results: Treated animals displayed a dose- and time-dependent increase in anxiety-like behavior and severe ultrastructural changes in neurons, astrocytes, and capillaries in both brain regions. Immunohistochemistry displayed astrogliosis with altered cell morphology. TNFα, IL-6, MDA, and CAT activity were significantly altered, depending on brain region and time post exposure. (4) Conclusions: AgNPs-CM induced neurobehavioral changes and severe cell lesions that continued to escalate after cessation of exposure.

## 1. Introduction

The exponential growth of the nanotechnology field has unlocked numerous and unique opportunities for improving the treatment and diagnosis of diseases. Nanotechnology involves analyzing, modeling, and manipulating nanostructures with diameters that range in size from 1 to 100 nm [1]. Depending on their shape, density, volume, and size distribution, nanostructures display novel optoelectronic, magnetic, and physiochemical properties [2]. Given their minuscule size, nanoparticles can enter and interact with structures and molecules within cells without causing damage. Based on this unique characteristic, nanostructures present numerous biomedical applications, including DNA labeling, drug delivery, gene delivery [3], immunotherapy, and potential for the development of novel medical materials and devices [4].

Silver nanoparticles (AgNPs) in particular have risen in popularity over the past decade. Due to their potent antimicrobial properties, they have been utilized with great success in both medicine and industry [5]. They have been extensively used as antimicrobial additives in fabric, laundry detergents, toothpaste, packaging, and cosmetics, as well as wound dressings, antiseptic sprays, and catheter coatings [5]. Additionally, AgNPs have been used as a basis for creating nanocomposites that build upon the antimicrobial properties of these nanoparticles, further enhancing the effectiveness and durability of their bactericidal effects [6]. Their small size, combined with their ability to absorb non-ionizing and ionizing radiation, has proven a great asset in increasing the effectiveness of oncology treatments, such as radiotherapy, photodynamic therapy [7], and photothermal therapy, as well as providing new options for monitoring of treatment efficiency [8]. 

The increased utilization and popularity of AgNPs has led to increased environmental and human exposure. Along with the continuous exploration of their potential benefits and applications, their long-term systemic effects must be thoroughly investigated and understood. While their mechanism of toxicity has not yet been conclusively defined, a great number of studies have demonstrated that AgNPs exhibit cytotoxic effects that vary according to their shape, size, charge, concentration, and coating agents but also according to targeted organisms, tissues, and cells. They decrease cell viability through the production of reactive oxygen species (ROS), signal-pathway interference, disruption of the cell cycle, cell-membrane disruption, and mitochondrial dysfunction, culminating in DNA damage and apoptosis [9,10]. As these toxic effects have most frequently been observed in AgNPs synthesized through chemical and physical methods, biological synthesis (also known as green synthesis) has emerged as a possible solution to the plethora of detrimental organic effects pf AgNPs. As opposed to classical methods, biological methods of nanoparticle synthesis, such as phytosynthesis, utilize molecules produced by polyphenol-rich plant extracts, bacteria, algae, and fungi as stabilizing and reducing agents [11]. By taking advantage of the high biocompatibility and powerful antioxidant compounds of plants, this eco-friendly method of synthesis directly affects the attributes of metal nanoparticles, stabilizing and ultimately diminishing their toxicity [12].

Biosynthesized AgNPs have been shown to accumulate in the brain, testes, liver, thymus, spleen, kidneys, lungs, stomach, bladder, and heart, regardless of route of administration [13]. Although most organs can efficiently remove AgNPs over time, this process is impeded and is significantly slower in the brain and testes than other organs [14]. In addition, it has been previously theorized that due to their slow nanoparticle clearance, AgNPs continue to exert cytotoxic effects for a prolonged period following interruption of exposure [15]. Along with the accumulation of nanoparticles at these sites, dose- and size-dependent toxicological effects have been documented, especially following administration of medium- and small-sized nanoparticles synthesized through chemical techniques. Metal nanoparticles have been documented to induce alterations in the brain, which include blood-brain-barrier (BBB) destruction and increased permeability, swollen astrocytes, neuronal degeneration, myelin-sheet abnormalities, synaptic degeneration, hippocampal apoptosis, and activation of glial cells and conversion into inflammatory cells [16]. It must be noted that the metal nanoparticles used in the majority of previous studies were obtained through chemical synthesis. Hence, there exists a real need to quantify the level to which biosynthesis can diminish the neurotoxicity of AgNPs.

*Cornus mas* L. (CM) is a wild plant belonging to the Cornaceae family that can be found growing across Asia and Europe. Its ripe scarlet fruits are rich in powerful antioxidant compounds (ascorbic acid, anthocyanin, polyphenol compounds, and flavonoids) and were traditionally used to treat numerous afflictions, from gastrointestinal disorders, urinary tract infection, and kidney stones to cancer and malaria. Studies on the effects of CM fruit extracts in experimental models of inflammation have confirmed their ability to significantly reduce lipid peroxidation and malondialdehyde (MDA) levels [17]. In addition, they have also been shown to exert various degrees of hypolipidemic, antimicrobial, and anticarcinogenic effects [18]. These properties make CM an ideal candidate for phytosynthesis of silver nanoparticles. 

The aim of the present study was to evaluate the effects of AgNPs phytosynthesized with *Cornus mas* extract (AgNP-CM) on the morphology and ultrastructure of the healthy rat brain in conjuncture with cognitive function, oxidative stress, and inflammation. Furthermore, as the majority of papers that investigated the effects of nanoparticles on brain tissue were conducted immediately after the animals were sacrificed, it is important to uncover whether any observed cytotoxic effects diminish, linger, or escalate following interruption of exposure to AgNPs. 

## 2. Materials and Methods

### 2.1. Reagents

The following reagents were purchased and used in the present study: osmium tetroxide, SDS, 2,4-dinitro-phenylhydrazine, Triton X-100, protease inhibitor complex Bradford, 2-thiobarbituric acid, Igepal-nonidet, and Folin Ciocalteu from Sigma-Aldrich Chemicals GmbH (Hamburg, Germany); hydrogen peroxide, ethanol, n-butanol, KH_2_PO_4_, 3H_2_O, K_2_HPO_4_, and Na_2_SO_4_ from Chimopar (Bucharest, Romania); NaOH, acetone, EDTA-Na_2_, uranyl acetate and silver nitrate (AgNO_3_) from KGaA (Darmstadt, Germany); lead citrate, and Epon 812 resin from Fluka GmbH (Buchs, Switzerland); and glutaraldehyde from Electron Microscopy Sciences (Hatfield, PA, USA).

### 2.2. Fruit-Extract Preparation

*Cornus mas* L. fruits were acquired in August 2019 from a farmers’ market in the city of Aiud, Alba County, Romania. The fresh, fully ripened Cornelian cherry fruits were cleaned of impurities by washing under cold tap water several times, followed by washing with deionized water. The fruit pulp was separated from the stones and manually crushed in a mortar. 10 g of crushed pulp paste was mixed with 200 mL distilled water in an Erlenmeyer flask. The mixture was kept at room temperature under constant stirring for 2 h and then centrifuged at 10,000× *g* rpm for 15 min. Following centrifugation, the supernatant was separated. The obtained *Cornus mas* fruit extract was stored at 4 °C in a dark container, away from sunlight, until synthesis of the silver nanoparticles. 

### 2.3. Biosynthesis and Characterization of Silver Nanoparticles

The biomolecules from Cornelian cherry fruit extract were used as reducing and capping agents for AgNP phytosynthesis. The silver ions from AgNO_3_ were reduced to their metallic form by adding 100 mL fruit extract dropwise into a 300 mL aqueous solution of 10^−3^ M of silver salt under vigorous stirring. The pH of the mixture was adjusted to 8.5 using NaOH solution (0.1 M). The bioreduction reaction started within a few minutes, as indicated by a significant color change from faint red to dark brown, confirming the formation of AgNPs. After 30 min, the reaction was complete, and the obtained colloidal solution was further centrifuged at 10,000 rpm for 30 min to separate the AgNP pellet. The pellet was washed several times with deionized water and then resuspended in deionized water for further analysis [19]. 

The biomolecule-capped AgNPs were characterized using several techniques that involved microscopy and spectroscopy. Bioreduction of the silver metal ions into silver nanoparticles and the surface plasmon resonance (SPR band) of the biosynthesized AgNPs were monitored by UV-Vis spectroscopy using a Perkin-Elmer Lambda 25 UV-Vis double-beam spectrophotometer (Perkin-Elmer, Boston, MA, USA) with 1 cm quartz cuvettes. The role of the phytocompounds from the fruit extract in the synthesis and stabilization of the AgNPs was assessed using Fourier transform infrared spectroscopy (FTIR) on a JASCO spectrophotometer (Easton, MD, USA). The particle size and morphology of the biomolecules-capped AgNPs were analyzed by transmission electron microscopy using an H-7650 120 kV Automatic TEM Hitachi (Tokyo, Japan) on a carbon-coated copper grid.

### 2.4. Animal Groups and Experimental Design

A total of 36 healthy, adult, male Wistar rats (10-week-old, 250–260 g body weight) were included in the present study. The animals were acquired from the Animal Department of ‘Iuliu Hatieganu’ University of Medicine and Pharmacy, Cluj-Napoca City, Cluj County, Romania. Appropriate housing and feeding conditions were ensured for the rats, animals had unrestricted access to water and food (VRF1 normo-caloric diet), were provided with 12 h light and dark cycles, and their environment was kept at a constant temperature of 25 °C and 35% humidity. Based on treatment, the rats were randomly assigned to three groups (n = 12 rats/group). Control group (ctrl) received normal saline, 1st test group (D1) received a 0.8 mg/kg body weight dose of AgNP-CM, and the 2nd test group (D2) received a 1.5 mg/kg body weight dose. All treatments were administered orally (by gavage) in a 0.5 mL saline solution. The treatment period lasted 45 days, with treatment administered daily, at approximately the same hour. Animals were sacrificed via cervical dislocation. Six rats of each group were sacrificed immediately after completion of treatment (T45), while the remaining six rats per group were sacrificed 15 days after the treatment ended (T60), the time in which the animals were allowed to recuperate. Post-treatment, rats from all groups were kept under the same conditions, with no change in diet and no medication administered. Before sacrificing the animals, their general locomotor activity and anxiety-like behavior were tested with the aid of the open field test (OFT) and elevated plus maze (EPM). Following sacrifice, the brain was removed and processed so as to assess oxidative stress markers and inflammatory cytokine levels and to perform histopathological and electron microscopy examinations of the frontal lobe and hippocampus. 

All procedures were approved by the Ethics Committee of ‘Iuliu Hatieganu’ University of Medicine and Pharmacy (No. 25/13.01.2017) in accordance with the European Convention for the Protection of Vertebrate Animals used in Experimental and Scientific Purposes, Council of Europe No. 123, Strasbourg 1985.

### 2.5. Behavioural Testing

#### 2.5.1. Open Field (OF) Test

Rats from different experimental groups were tested in the open field two days prior to sacrifice, on days 43 and 58 of the experiment. The open field used was a rectangular arena (100 × 100 × 40 cm) divided electronically into 9 equal squares. Rats were introduced in the center of the arena and were allowed to explore the OF for a period of 5 min. After the allotted time, the rats were carefully removed and returned to their cage. Between subjects, the arena was disinfected with 70% ethanol. The activity of the animals was recorded and quantified with the aid of a visual tracking system (Smart Basic Software version 3.0 Panlab Harvard Apparatus). The arena was subdivided into 2 areas: central and peripheral. The general locomotion index of the animals was measured by the following parameters: total and peripheral traveled distance, total and peripheral numbers of entries. Further, distance traveled in the central area, number of entries in the central area, as well as the time ratio (time spent in central area/total time in the arena) were considered markers of low levels of anxiety-like behavior [20].

#### 2.5.2. Elevated Plus Maze

The EPM test was used to measure and quantify the anxiety-like behavior of the rats included in the study. The test was performed on days 44 and 59 of the experiment (one day before animals were sacrificed). The EPM apparatus was comprised of a plus-shaped maze with two open (10 × 50 cm) and two closed (10 × 50 × 40 cm) arms. The entire maze was elevated 60 cm above ground level. Initially, rats were placed in the center of the maze and turned towards one of the open arms, after which they were allowed to explore the maze for 5 min. Between subjects, the arena was disinfected with 70% ethanol. The activity of the animals was recorded and quantified with the aid of a visual tracking system (Smart Basic Software version 3.0 Panlab Harvard Apparatus). The motor activity of the animals was measured by traveled distance in closed arms, number of entries in closed arms, and number of total entries. To evaluate anxiety-like behavior, the following parameters were assessed: traveled distance in open arms, the number of entries in open arms, and time ratio (time spent in open arms/total time spent in the maze) [20].

### 2.6. Oxidative-Stress Assessment

In order to assess oxidative stress, MDA levels and CAT activity in the frontal lobe and hippocampus of rats were analyzed. Tissue homogenates were obtained, and CAT activity was investigated, as previously described [21], and expressed as units/g protein. MDA level was determined utilizing the fluorimetric method with 2-thiobarbituric acid, as previously described [22], and expressed as nmol/mg protein. 

### 2.7. Transmission Electron Microscopy

Immediately after the animals were sacrificed, samples of the hippocampus and frontal lobe (2 mm^3^) were collected from each animal and fixed with 2.7% glutaraldehyde in 0.1 M PBS (pH = 7.4) for 1.5 h at 4 °C. The fixative agent was then washed with the same buffer (3 × 1 h, and one overnight), and the samples were further post-fixed with 1.5% OsO_4_ in 0.15 M PBS for 1.5 h at 4 °C. Serial dehydration was performed with increasing concentrations of acetone (30–100%). Further, samples were infiltrated with Epon 812 solutions in acetone and then embedded in 300 μL capsules (Electron Microscopy Sciences, Hatfield, PA, USA). Lastly, the resin was polymerized for 72 h at 60 °C. The samples were cut into 60–80 nm sections with a DiATOME diamond knife (DiATOME, Hatfield, PA, USA) on an LKB Ultrotome III Bromma 8800 ultramicrotome (LKB Produckter AB, Stockholm-Bromma, Sweden). Sections were collected on 300 mesh Cu grids (Agar Scientific Ltd., Stansted, UK) and double-contrasted with a saturated solution of uranyl acetate (15 min) and 2.8% lead citrate solution (5 min). Sections were examined with a JEOL JEM 1010 microscope (Jeol Ltd., Tokyo, Japan) operating at 80 kV. Photographs of images were captured with a Mega VIEW III system (Olympus, Soft Imaging System, Munster, Germany).

### 2.8. Immunohistochemistry and Histology

As soon as the rat brain was removed and samples for TEM were taken, it was immediately fixed for 24 h in a 10% phosphate-buffered formalin solution. Following paraffin-wax embedding, 5 μm sections were cut with a Reichert microtome (Vienna, Austria) and stained with hematoxylin and eosin. Analysis of the slides was performed by two individual specialists (from the Institute of Biological Research, Cluj-Napoca, Romania) who had no previous knowledge of the experimental design. The slides were investigated with the aid of an Olympus BX51 microscope (Tokyo, Japan).

After tissue of interest was embedded in paraffin, 5 µm sections were cut and mounted onto glass slides that had been electrostatically charged. Dewaxing of tissue sections was performed with xylene, followed by rehydration. To achieve antigen retrieval, slides were incubated for 10 min with citrate buffer (pH = 6) at 95 °C. Further, the sections were exposed for 10 min to 3% hydrogen peroxide and then washed with PBS (0.01 M pH = 7.4). To prevent nonspecific background staining, slides were then treated with 10% BSA in PBS (pH = 7.4) for 1 h. The sections were then incubated at 4 °C for 12 h with anti-glial fibrillary acidic protein (GFAP) monoclonal antibody (SAB5201104, Sigma Aldrich Chemicals GmbH, Sternheim, Germany) (1:350 dilution). After incubation, sections were washed with PBS and treated with biotinylated-HRP link universal (Dako North America Inc., Carpinteria, CA, USA, LSAB + System-HRP) and left at room temperature for 15 min. Following another wash with PBS, sections were treated with streptavidin-peroxidase (Dako North America Inc., Carpinteria, CA, USA) and left to incubate for 15 min. A final wash with PBS was performed, followed by a 5-min incubation with DAB (DAB + Chromogen, Dako North America Inc., Carpinteria, CA, USA). The final step included counter-staining with Mayer’s hematoxylin and washing the slides in absolute ethanol (3 times) and xylene (one time) before covering the slides with synthetic resin. A positive reaction was identified as a brown coloration of astrocytes (body and processes). Negative controls were performed utilizing the same steps presented previously, with the difference that the primary antibody was swapped out for phosphate-buffered saline.

### 2.9. Statistical Analysis

Statistical analyses of data were performed using GraphPad Prism software, version 6.0 (GraphPad, San Diego, CA, USA). The results were expressed as mean ± SD of the mean. Statistical significance among the groups was determined by using one-way analysis of variance (ANOVA), followed by Tukey’s post hoc test. A *p*-value < 0.05 was considered statistically significant.

## 3. Results

### 3.1. Synthesis and Characterization of Silver Nanoparticles

**Figure 1:**
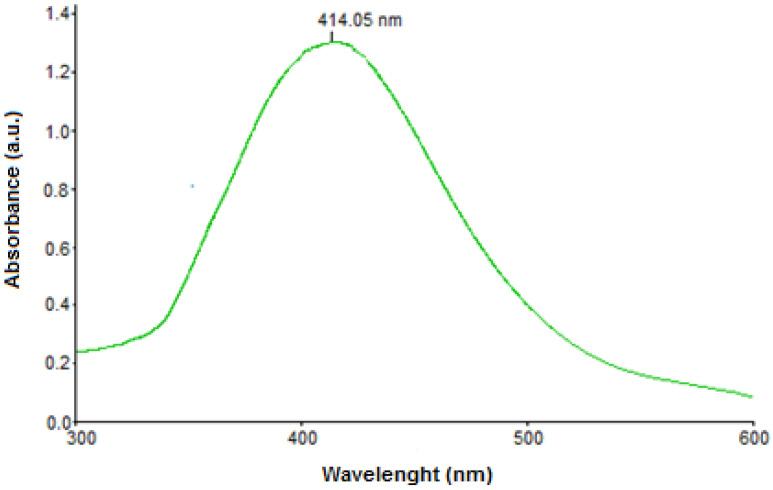
UV-Vis spectrum of AgNPs.

Plant-mediated synthesis of silver nanoparticles was achieved by exploiting the antioxidant capacity of biomolecules from Cornelian cherry fruit extract. The fruit’s phytocompounds were responsible for the reduction of silver ions into the corresponding metallic nanoparticles, as well as for the stabilization of the obtained colloid by preventing self-aggregation. The rapid change of reaction-mixture color clearly indicated the formation of AgNPs, which was also confirmed by recording the UV-Vis spectrum. The intense SPR band at 414 nm, as depicted in Figure 1, confirms the formation of the desired nanoparticles, as it is the characteristic surface plasmon resonance of metallic silver, which normally ranges between 400 and 450 nm [23,24,25].

**Figure 2:**
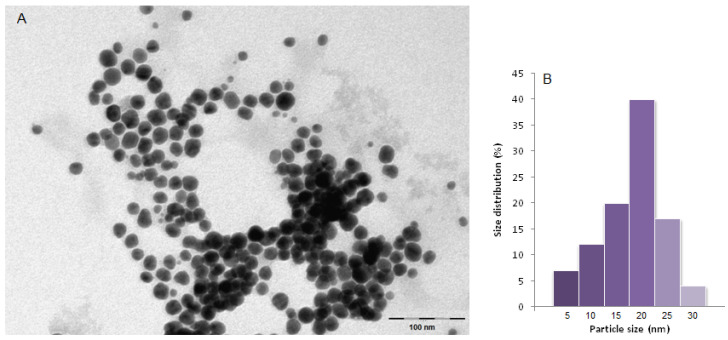
(**A**) TEM image of green synthesized silver nanoparticles and (**B**) size distribution.

The morphology and size of the biomolecule-capped AgNPs were investigated by transmission electron microscopy (TEM). The image (Figure 2) revealed that the obtained nanoparticles are spherical in shape, ranging in size from 5 nm to 30 nm, with the highest percentage size distribution at 20 nm in diameter (39%), and highly dispersed in solution, confirming the ability of the fruit biomolecules to act as stabilizing agents of AgNPs.

**Figure 3:**
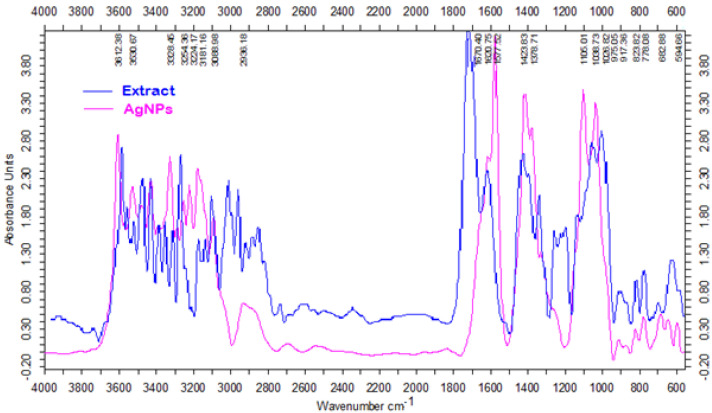
FTIR spectra of Cornelian cherry fruit extract and green synthesized AgNPs.

FTIR spectroscopy was also used to confirm the presence and chemical nature of the biomolecules at the surface of the silver nanoparticles. It is well known that *Cornus mas* fruits are particularly rich in phenolic compounds [26], especially anthocyanins and flavonoids [27]. These biomolecules are known to possess high antioxidant activity and were expected to act as reducing and capping agents of the synthesized AgNPs. Figure 3 shows the FTIR spectra of Cornelian cherry fruit extract and green synthesized silver nanoparticles, clearly confirming the presence of these compounds at the surface of the AgNPs.

The strong absorption peaks present in the range of 3254–3606 cm^−^^1^ in the spectrum of Cornelian cherry extract can be assigned to OH stretching in phenols and alcohols. The stretching vibration of the C=O bond from the aldehydes, ketones, or organic acids has generated a peak at 1730 cm^−1^, while the C–O stretching vibration in phenolics resulted in a peak at 1267 cm^−1^. The spectrum of the phytosynthesized AgNPs reveals the presence of the same functional groups, with the characteristic peaks of these groups slightly shifted, e.g., the OH stretching vibration appears in the range of 3328–3612 cm^−1^, while the C=O vibration generated a peak at 1577 cm^−1^, confirming the stabilization capacity of the carboxylic acids, phenolic compounds, and flavonoids from the fruit extract, which prevent aggregation of the AgNPs. The extract FTIR spectrum showed major peaks that were consistent with OH stretching of phenols and alcohols with strong hydrogen bonds. Due to the stretching vibration of the C=O functional group from ketones, aldehydes, or organic acids, a band at 1730 cm^−^^1^ was observed. Likewise, the peak at 1267 cm^−^^1^ can be ascribed to the C–O stretching vibration in phenolic compounds. Regarding the biosynthesized AgNPs, the FTIR spectrum reveals the absence of the characteristic peak of the carbonyl group, thus demonstrating the involvement of extract biomolecules containing these groups in the reduction of silver ions. The FTIR analysis also indicated a shift of the absorption peak of the hydroxyl group, which now appears at 3612 cm^−^^1^. The ζ-potential of the silver nanoparticles synthesized with *Cornus mas* fruit extract was −31 mV [15]. 

### 3.2. Behavioural Tests

#### 3.2.1. Open Field Test

**Figure 4:**
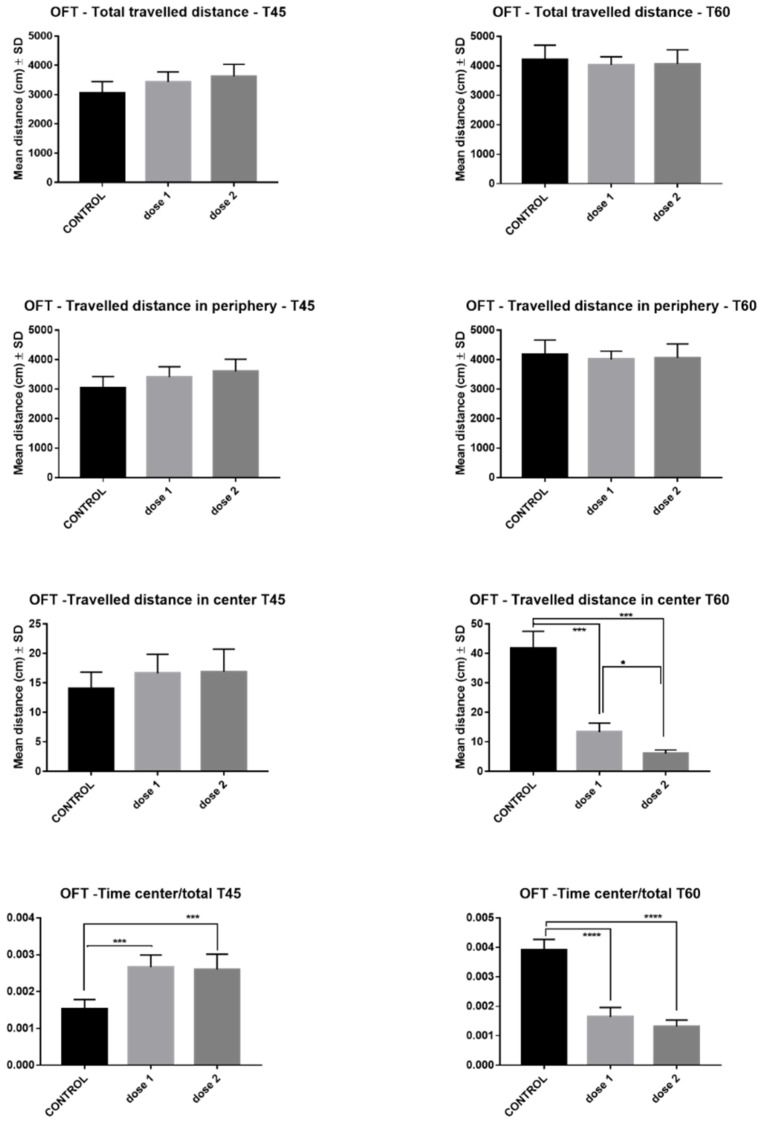
Open field test results–comparison between control, low-dose (D1), and high-dose (D2) groups in rats sacrificed immediately after end of treatment (T45) and those sacrificed 15 days post-treatment (T60). * *p* < 0.05; *** *p* < 0.001; **** *p* < 0.0001.

The effects of AgNPs-CM on rat locomotion and anxiety tested in OFT are illustrated in Figure 4. AgNP administration did not affect total travelled distance or the traveled distance in periphery in either treated group at T45 and T60. However, regarding traveled distance in center at T45, a slight dose-dependent increase was noted, while at T60, a significant reduction was observed in D1 (*p* < 0.0001) and D2 (*p* < 0.0001). The time spent in center/total time spent in labyrinth was significantly increased in D1 (*p* < 0.001) and D2 (*p* < 0.001) at T45, while at T60, this ratio was markedly decreased in both treated groups (*p* < 0.0001).

#### 3.2.2. Elevated Plus Maze

**Figure 5:**
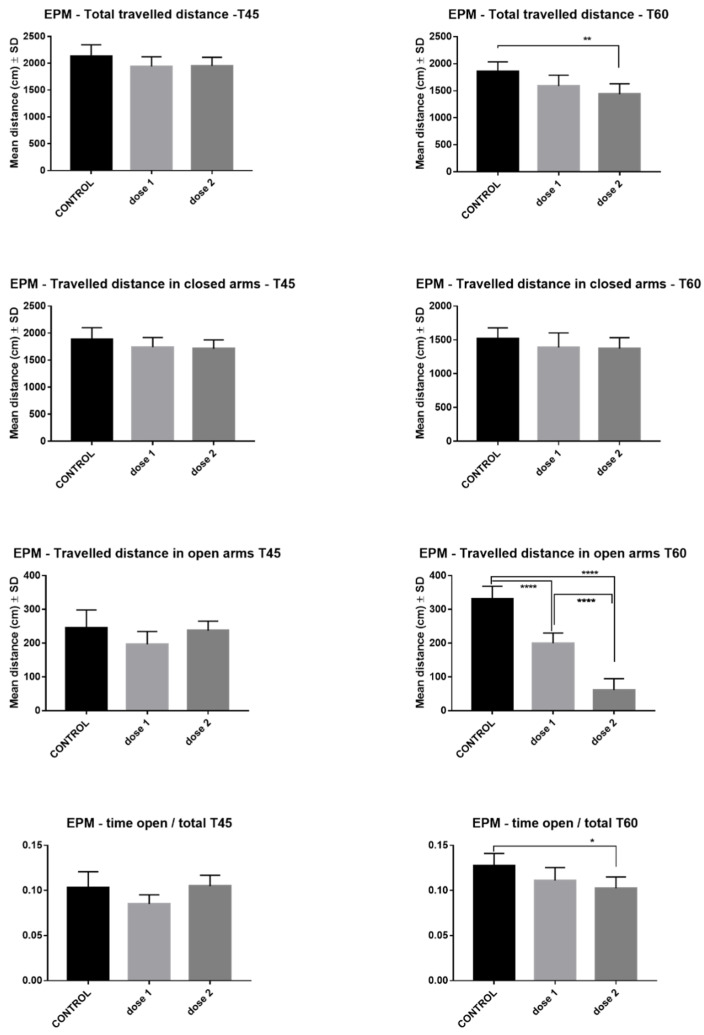
Elevated Plus Maze—comparison between control, low-dose (D1), and high-dose (D2) groups in rats sacrificed immediately after end of treatment (T45) and those sacrificed 15 days post-treatment (T60). * *p* < 0.05; ** *p* < 0.01; **** *p* < 0.0001.

The effects of AgNPs-CM on rat locomotion and anxiety tested in EPM are illustrated in Figure 5. Total traveled distance at T45 presented no modification in treated groups compared with controls, while at T60, the total traveled distance significantly decreased, especially in the D2 group (*p* < 0.001). At T45 and T60, both treated groups had traveled similar distances in closed arms compared to controls. Both traveled distance in open arms (D1, D2, *p* < 0.001) and time ratio (open arms/total time) (D2, *p* < 0.05) significantly decreased in a dose-dependent manner at T60 (*p* < 0.0001). The above-mentioned differences were not recorded at T45.

### 3.3. Oxidative-Stress Asessement

**Figure 6:**
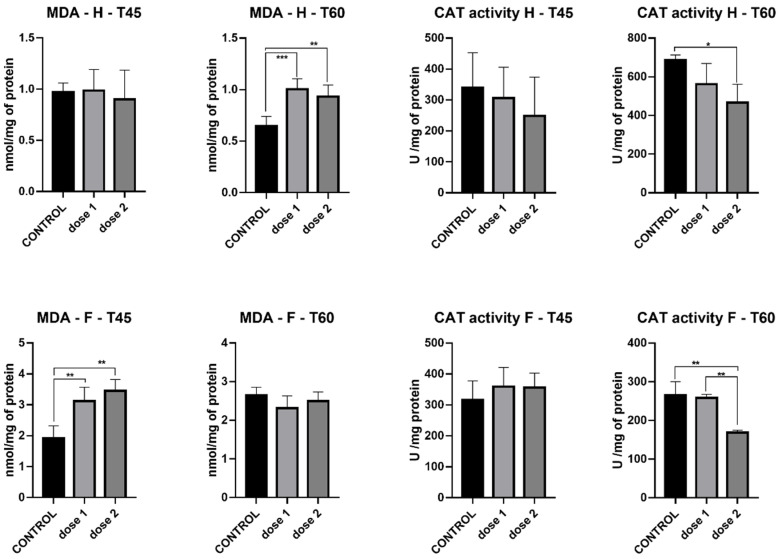
Malondialdehyde levels and catalase activity in the hippocampus (H) and frontal cortex (F) of groups sacrificed immediately after treatment (T45) and those sacrificed 15 days post-treatment (T60). * *p* < 0.05; ** *p* < 0.01; *** *p* < 0.001.

MDA levels in the hippocampus were stable between the 2 different doses and control at T45. However, at T60, a marked increase in MDA levels was recorded in D1 (*p* < 0.001) and D2 (*p* < 0.01). It must be noted that although the increase is statistically significant, total MDA was similar between treated groups (D1 and D2) at T45 and T60. MDA levels in the frontal lobe present an opposite trend, with significant increases in both treated groups at T45 (*p* < 0.01) but no difference at T60 between control and treatment groups (Figure 6). 

Catalase activity measured in the hippocampus presented a decrease in both treatment groups compared to control, with significant a difference present only at T60; D2 (*p* < 0.05). In the frontal lobe, at T45, catalase activity was similar between control and treatment groups, while at T60, a marked decrease was registered in D2 (*p* < 0.01) (Figure 6). 

**Figure 7:**
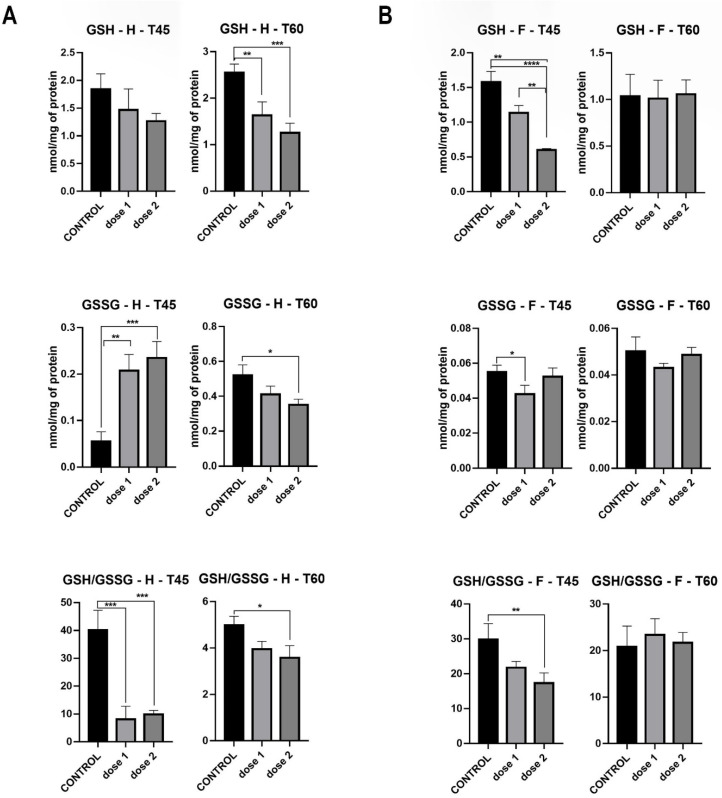
GSH and GSSG levels, GSH/GSSG ratio in hippocampus (**A**) and frontal lobe (**B**) of groups sacrificed immediately after treatment (T45) and those sacrificed 15 days post-treatment (T60). * *p* < 0.05; ** *p* < 0.01; *** *p* < 0.001; **** *p* < 0.0001.

GSH levels measured in the hippocampus showed a dose-dependent drop at T60, where statistical significance was observed in D1 (*p* < 0.01) and D2 (*p* < 0.001). GSSG levels at T45 were markedly higher at D1 (*p* < 0.001) and D2 (*p* < 0.001) compared to control, whereas at T60, a dose-dependent decrease was observed, with statistical significance in D2 (*p* < 0.05). The GSH/GSSG ratio significantly decreased in both treated groups (*p* < 0.001) at T45, while at T60, only a slight yet statistically significant reduction was observed in D2 (*p* < 0.05) (Figure 7). 

GSH levels measured in the frontal lobe showed a significant dose-dependent drop (D1: *p* < 0.001 and D2: *p* < 0.0001) at T45, which did not remain constant at T60, where levels evened out between treated and control groups. GSSG levels at both T45 and T60 showed no statistical difference between control and treated groups. The GSH/GSSG ratio followed the same trend, with a dose-dependent reduction at T45 (D2: *p* < 0.05) and a uniformization between control and treated groups at T60 (Figure 7). 

### 3.4. Pro-Inflammatory Cytokines 

**Figure 8:**
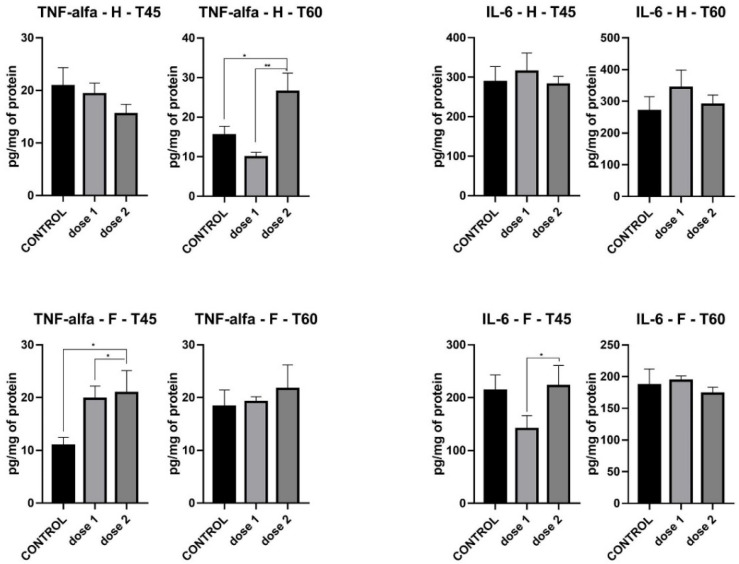
IL-6 and TNF-alpha levels in hippocampus (H) and frontal cortex (F) of groups sacrificed immediately after treatment (T45) and those sacrificed 15 days post-treatment (T60). * *p* < 0.05; ** *p* < 0.01.

There was no significant difference in hippocampus IL-6 levels between control and treated groups at both T45 and T60. In the frontal lobe, IL-6 secretion had the same pattern at T60 as that observed in the hippocampus, while at T45, a slight decrease was observed in D1 (*p* < 0.05) (Figure 8). 

TNF-alpha in the hippocampus did not register any modification between control and treated groups at T45, while at T60, a sharp increase was found in D2 compared to both D1 (*p* < 0.01) and control (*p* < 0.05) groups. In the frontal lobe, TNF-alpha levels at T45 recorded a significant increase in both D1 and D2 (*p* < 0.05), levels which evened out at T60, where no statistical difference between control and treated groups was found (Figure 8). 

### 3.5. Tranmission Electron Microscopy

**Figure 9:**
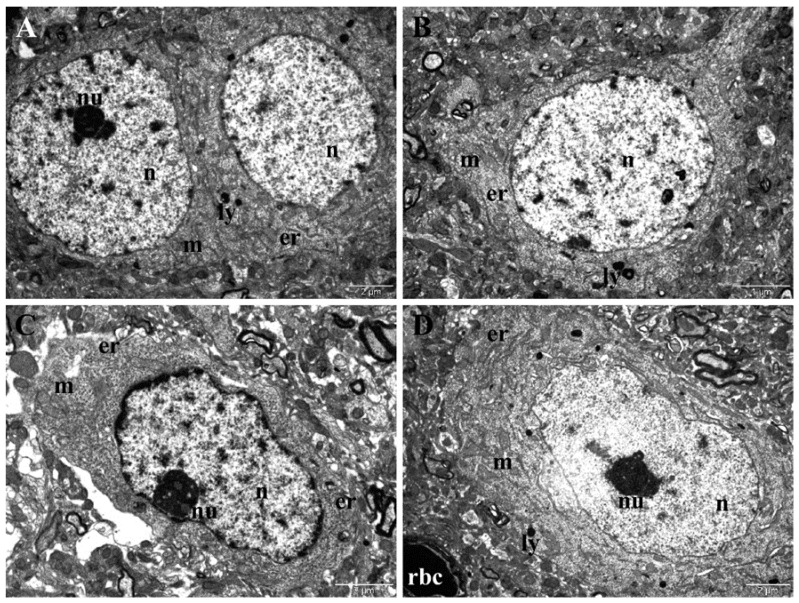
(**A**,**B**) TEM images showing normal ultrastructure of neurons in the frontal cortex of control group. (**C**,**D**) TEM images showing normal ultrastructure of neurons in the hippocampus (**C**,**D**) of control group. Er–endoplasmic reticulum; ly–lysosome; m–mitochondrion; n–nucleus; nu–nucleolus; rbc–red blood cell.

TEM examination of samples collected from the control group showed normal ultrastructure of the neurons both in the frontal cortex and in the hippocampus. The neurons in the frontal cortex contained round-oval and euchromatic nuclei, elongated mitochondria, abundant endoplasmic reticula, and few lysosomes (Figure 9). The neurons in the hippocampus had oval, euchromatic nuclei, with a slightly irregular contour and prominent nucleoli, an important amount of endoplasmic reticulum, many oval mitochondria, and rare lysosomes (Figure 9C,D). 

**Figure 10:**
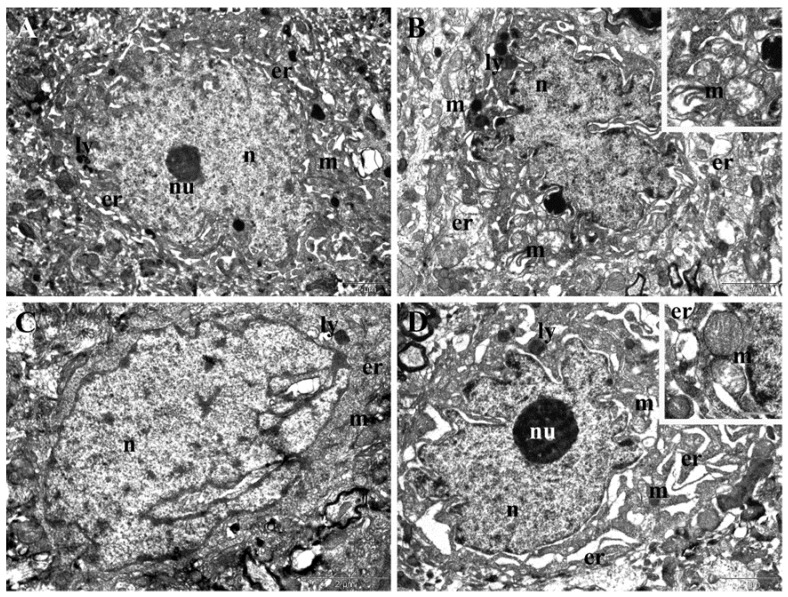
TEM images presenting ultrastructural changes recorded at the end of the experimental treatment. (**A**) Neurons from frontal cortex of rats treated with the low dose of NPs (T45 D1 group). (**B**) Neurons from the hippocampus of rats treated with the low dose of NPs (T45 D1 group). (**C**) Neurons from the frontal cortex of rats treated with the high dose of NPs (T45 D2 group). (**D**) Neurons from the hippocampus of rats treated with the high dose of NPs (T45 D2 group). er—endoplasmic reticulum; ly—lysosome; m—mitochondrion; n—nucleus; nu—nucleolus; rbc—red blood cell.

TEM examination of samples collected in the last day of treatment from the animals experimentally treated with the two doses of NPs revealed important ultrastructural changes in the neurons from the frontal cortex and hippocampus. In the frontal cortex, administration of the low dose of NPs in the T45 group was followed by changes in the shape of nuclei, which became irregular, and of endoplasmic reticula, the lumen of which was enlarged in some neurons (Figure 10A). The hippocampal neurons in the T45 D1 group displayed nuclei with highly altered shape and enlarged perinuclear space, dilated endoplasmic reticula, and mitochondria with rarefied matrices and altered cristae (Figure 10B). The frontal cortex of the T45 D2 group presented accentuated nuclear polymorphism and slight dilatation of endoplasmic reticula (Figure 10C). The hippocampal neurons in the T45 D2 group displayed nuclei with a tendency of lobation, very enlarged perinuclear space, and prominent nucleoli in many cases. The endoplasmic reticulum was proliferated and extremely dilated, while mitochondria contained rarefied matrices and altered cristae (Figure 10D). Additionally, hippocampus and frontal cortex neurons of both groups (T45 D1 and T60 D2) presented an increased number of lysosomes surrounding the nucleus (Figure 10A,B). 

**Figure 11:**
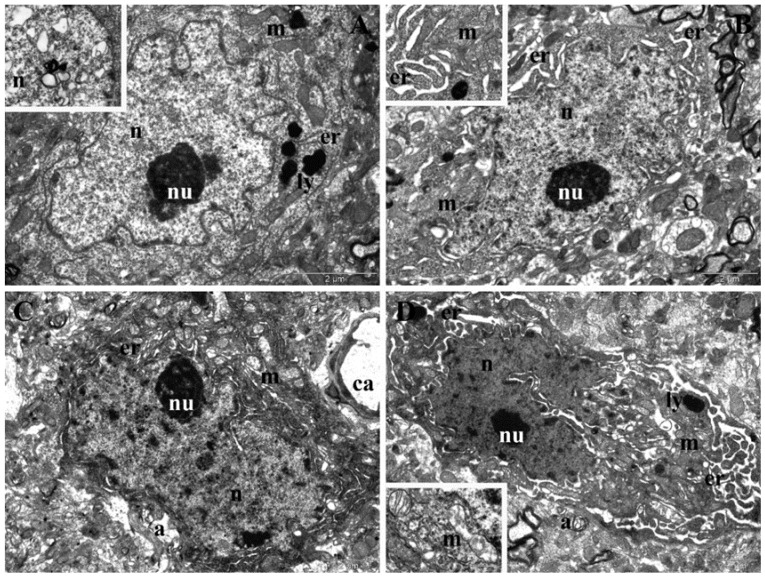
TEM images presenting ultrastructural changes recorded 15 days after the end of the experimental treatment. (**A**) Neurons from the frontal cortex of rats treated with the low dose of NPs (T60 D1 group). (**B**) Neurons from the hippocampus of rats treated with the low dose of NPs (T60 D1 group). (**C**) Neurons from the frontal cortex of rats treated with the high dose of NPs (T60 D2 group). (**D**) Neurons from the hippocampus of rats treated with the high dose of NPs (T60 D2 group). a—autophagosome; ca—capillary; er—endoplasmic reticulum; ly—lysosome; m—mitochondrion; n—nucleus; nu—nucleolus; rbc—red blood cell.

On the TEM samples collected 15 days after the experimental treatment, more accentuated ultrastructural changes were found in the neurons from the frontal cortex of the animals in the T60 D1 group, represented mainly by nuclear and mitochondrial polymorphism (Figure 11A). However, a striking degenerative alteration was represented by extensive lesions of chromatin in many neurons (inset of Figure 11A). In the hippocampus of the T60 D1 group, we found neurons with highly irregular shape and enlarged perinuclear space, moderately dilated endoplasmic reticula, and polymorphous mitochondria (Figure 11B). In the frontal cortex of the T60 D2 group, we also noted accentuated nuclear polymorphism, enlarged perinuclear space, dilated endoplasmic reticula, numerous elongated mitochondria with rarefied matrices, mitochondria with electron-dense granules, as well as autophagosomes near the periphery of the nucleus (Figure 11C). Hippocampal neurons in the T60 D2 group had the highest degree of polymorphism, also displaying condensed chromatin and enlarged perinuclear space. The endoplasmic reticulum was also highly proliferated, and many mitochondria with altered cristae, rarefied matrices, and electron-dense granules were found. Autophagosomes were also observed (Figure 11D). Markedly large and irregular electron-dense lysosomes were observed in hippocampus and frontal-cortex neurons of both groups (Figure 10A,D). 

Normal ultrastructure of astrocytes and capillaries from the hippocampus and frontal cortex of the control group were presented (Supplementary Figure S1). 

In the frontal cortex of T45 D1 animals, we observed irregularly shaped capillaries and vacuolization and lysis of astrocyte end-foots (Supplementary Figure S2A). For the rats treated with the higher dose of NPs (T45 D2 group), the capillary endothelium appears to be proliferated and still irregular. Astrocyte end-foots were vacuolated and enlarged (Supplementary Figure S2B). Astrocytes of the T45 D1 group (Supplementary Figure S2C) presented with rarefied chromatin, enlarged nuclei, and large, oval mitochondria, while in the T45 D2 group, whole astrocytes appeared to be lysed, with free-floating mitochondria and nuclei (as observed in Supplementary Figure S2D). Hippocampal tissue of T45 groups was examined and is presented in Supplementary Figure S3. We observed similar changes to those in the frontal cortex, including proliferation of capillary endothelium and vacuoles in astrocyte end-foots in both T45 D1 and T45 D2 groups (Supplementary Figure S3A,B). The astrocytes themselves appear to be viable, with large, oval nuclei with increased amounts of condensed chromatin. Some mitochondria contained electron-dense granules, and the cells from the T45 D2 group appeared to have an enlarged perinuclear space (Supplementary Figure S3D). Furthermore, we noted more myelin fibers present than in the frontal-cortex samples. 

The frontal-cortex tissue of the T60 D1 group showed aberrant endothelial cells with large, polymorphous mitochondria (Supplementary Figure S4A). Astrocytes had large nuclei with increased amounts of heterochromatin coating the nuclear membrane. In the group treated with the higher dose of NPs (T60 D2 group), the lesions observed were similar, yet much more pronounced. Astrocyte end-foots surrounding the capillary presented autophagosomes, and some appeared to be lysed (Supplementary Figure S4C). In their perinuclear cytoplasm, mitochondria with electron-dense granules were observed. In both groups, the endoplasmic reticulum was nearly absent, and the cytoplasm itself was rarefied. The T60 D1 hippocampal tissues showed normal ultrastructure of the capillaries and astrocytes, with increased perinuclear space (Supplementary Figure S5A,C). In the T60 D2 group, capillary ultrastructure maintained a rather normal aspect, although in their vicinity, a higher degree of astrocyte end-foot vacuolization was observed. Astrocytes had smaller nuclei with more condensed chromatin and vacuolized cytoplasm. Mitochondria appeared enlarged with rarified matrices (Supplementary Figure S5B,D). 

### 3.6. Immunohistochemistry and Histology

**Figure 12:**
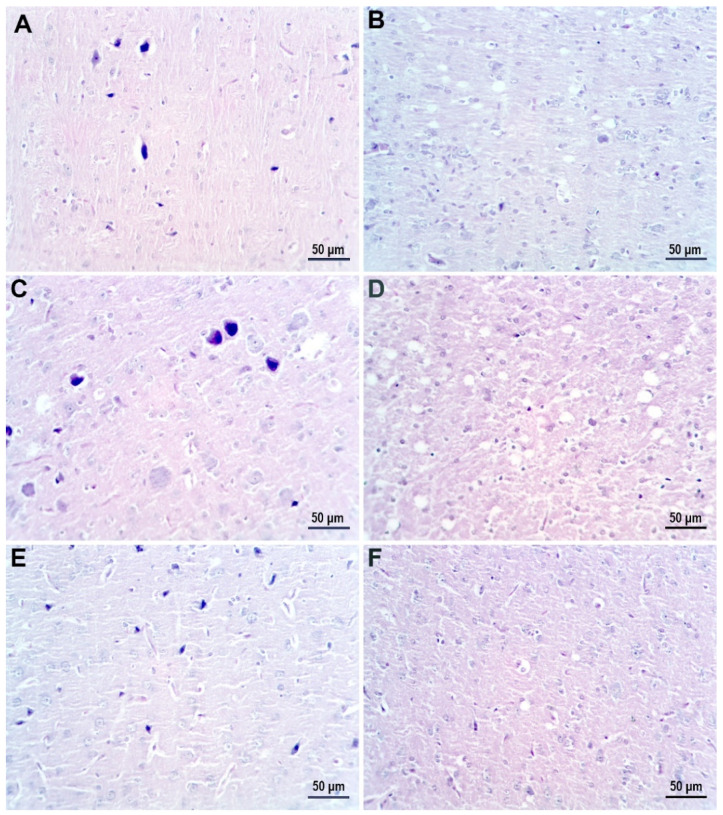
Hematoxylin-eosin-stained sections of cerebral-cortex tissue. (**A**,**B**) Control group displaying normal morphology. (**C**) Groups treated with low dose of AgNP and sacrificed immediately after treatment. (**E**) Groups treated with high dose of AgNP and sacrificed immediately after treatment. (**D**) Groups treated with low dose of AgNP and sacrificed 15 days post-treatment. (**F**) Groups treated with high dose of AgNP and sacrificed 15 days post-treatment.

Figure 12 presents light microscopy examination of hematoxylin-eosin-stained sections of cerebral-cortex tissue from the control group (Figure 12A,B) and AgNP-treated groups. In the T45 D1 and T45 D2 groups, large apoptotic neurons with pericellular edema were observed (dark neurons) (Figure 12C,E). In the T60 D1 and T60 D2 groups, the primary modifications observed were myelin vacuolization and neuropile with a heterogenous aspect, with the added distinction of karyorrhexis and pericellular edema in the T60 D2 group (Figure 12D,F).

**Figure 13:**
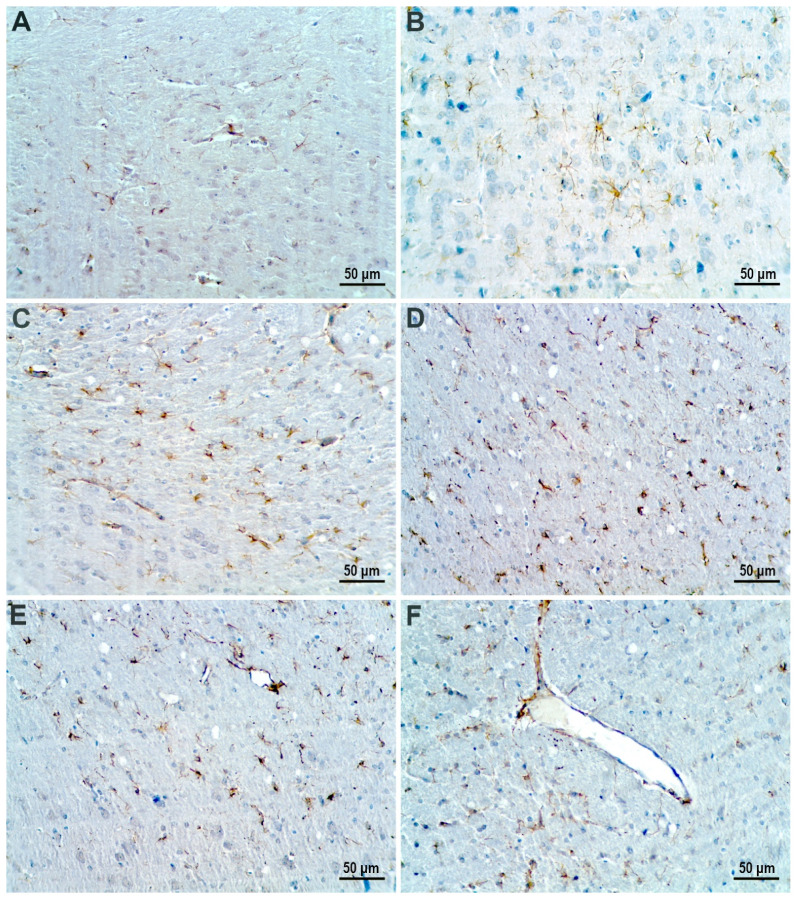
GFAP immune-stained sections of cerebral-cortex tissue. (**A**,**B**) Control group. (**C**) Groups treated with low dose of AgNP and sacrificed immediately after treatment. (**E**) Groups treated with high dose of AgNP and sacrificed immediately after treatment. (**D**) Groups treated with low dose of AgNP and sacrificed 15 days post-treatment. (**F**) Groups treated with high dose of AgNP and sacrificed 15 days post-treatment.

Cerebral cortex of the control group presented normal-sized and mature GFAP-positive astrocytes (Figure 13A,B). The AgNP-treated groups showed different degrees of astrogliosis. In the T45 D1 and T45 D2 groups, moderate astrogliosis manifested through astrocyte proliferation was observed. Astrocytes displayed a more intense expression for GFAP and, although present in larger numbers, they presented a smaller cellular body and a reduced length of processes (Figure 13C,E). In the T60 D1 and T60 D2 groups, astrogliosis was exhibited through a pronounced astrocyte proliferation with marked astrocyte hypotrophy. In addition to displaying a reduced number of processes, these were also extremely short (Figure 13D,F).

## 4. Discussion

In the present study, we have chosen to use a biological method of nanoparticle synthesis that takes advantage of the polyphenol-rich content of the *Cornus mas* fruit with the purpose of creating a highly biocompatible NP. The current commonly employed methods of nanoparticle synthesis consist of chemical and physical strategies that utilize hazardous solvents, reducing agents, and stabilizers. These substances have been shown to have undesirable toxic effects, such as carcinogenicity and environmental pollution [28], which greatly limit the applicability of NPs in the biomedical and clinical fields [29]. As an answer to these restrictions, biological methods have arisen as a clean, low-cost, and eco-friendly alternative to the classically used methods [30]. Biological methods of nanoparticle synthesis take advantage of the high biocompatibility and availability of bacteria, fungi, viruses, and plants, which act as stabilizing and capping agents for metal nanoparticles, directly influencing their size, shape, and physicochemical characteristics [31]. The decision to use plants as a base for the biological synthesis method was due to their lack of pathogenicity and greater concentration of antioxidant compounds compared to bacterial, fungal, and viral alternatives. *Cornus mas* fruit extract in particular has been shown to display powerful anti-oxidant and anti-inflammatory effects on in vivo rat models [17,19]. Following the addition of the fruit extract to AgNO_3_, nanoparticle formation was successfully completed in 30 min. The obtained AgNPs were spherical in shape, and size distribution of the obtained AgNPs was homogeneous, ranging from 5 to 30 nm. FTIR spectroscopy confirmed successful capping of the AgNPs through the presence of *Cornus mas*-derived phenolic compounds on their surface. Similar silver nanoparticles with a diameter ranging from 10–20 nm were obtained by Bar et al. by using *Jatropha curcas* extract [32], while Das et al. utilized *Nyctanthes arbor-tristis* flower extract to phytosynthesize spherical gold nanoparticles with an average diameter of 20 nm [33]. 

The AgNP-CM were administered orally, as this route of exposure is one of the most prevalent encountered by the general human population. Ingestion of AgNPs by humans has been shown to be facilitated by their presence in fresh and processed foods (migration of AgNPs from packaging, coatings on food-handling machinery, anti-spoilage AgNPs sprays), water, fish, and other aquatic animals (aquatic ecosystem contamination due to industrial and urban effluents) [34]. A previous report by the WHO estimated that 20–80 µg of Ag, including nanomaterials, is ingested by humans daily [35]. However, taking into consideration the ever-expanding use and incorporation of nanoparticles in everyday objects, this amount is most likely an underestimation. As demand for nanoparticles has risen, so has their utilization. In 2010, an estimated 20 tons of Ag nanomaterials were produced in the United States alone, while an estimated 450–542 tons were produced worldwide in 2014 [36]. In order to closely mimic human daily oral exposure and ingestion of AgNPs, two different doses were administered to the rats included in the present study: a lower dose of 200 µg/day (0.8 mg/kg body weight) and a higher dose of 375 µg/day (1.5 mg/kg body weight). These doses were also chosen so as to account for the difference in Ag absorption rates between rats (0.4–10%, depending on species) and humans (18%) [37]. 

One of the aims of the present study was to assess whether exposure to CM-biosynthesized AgNPs would affect the behavior of rats. For this purpose, OFT and EPM tests were conducted on animals included in the study. These two methods were chosen for their ability to evaluate rat emotionality (anxiety) and locomotion [38]. While it was clear that locomotion was not affected by the administration of silver nanoparticles, a dose-dependent and time-dependent increase in anxiety-like behavior was noted in the treated rats. In contrast to rats sacrificed immediately after administration of treatment, which expressed little to no anxiety-like behavior, regardless of AgNP dose, groups sacrificed 15-days post-treatment displayed a marked increase in anxiety levels. This was expressed in the two tests as a reduction in traveled distance in open arms (EPM), distance traveled, and time spent in center (OFT). Similar results were obtained by Antsiferova et al. [39] after prolonged oral administration of PVP-coated silver nanoparticles (50 µg daily, up to 180 days) to C57Bl/6 male mice. They observed that anxiety-like behavior was present after 30 to 60 days of exposure to AgNPs, after which the mice displayed research instinct and finally a degradation in memory. However, other studies that administered higher doses of silver nanoparticles observed neurobehavioral alterations in rats much sooner. One such study conducted by Javurek et al. [40] found that following daily oral administration of 3.6 mg/kg body weight AgNPs, rats displayed anxiety-like behavior after a period of two weeks. Interestingly, researchers found no significant histopathological modifications at the level of the brain or gastrointestinal system. Instead, the behavioral changes were correlated with a select reduction in bacterial intestinal flora. 

A second aim of the present study was to assess the effect of AgNPs-CM on the rat brain ultrastructure. At a cellular level, neurons reacted to the experimental conditions by degenerative alteration that concerned mainly the nuclei but also cell organelles, such as the endoplasmic reticulum and mitochondria. The recorded ultrastructural changes of neurons in the frontal cortex and hippocampus were similar and dose-dependent. In the groups sacrificed 15 days post-treatment (T60), the dose-dependent changes observed in the neurons from the frontal cortex were more prominent compared to those observed in experimental groups sacrificed immediately after treatment (T45). On the other hand, the degenerative alterations of neurons in the hippocampus were comparable between the two groups, especially at the levels of nuclei and mitochondria, though more accentuated in the hippocampus of T45 groups. However, hippocampus ultrastructural modifications in the T60 lower-dose (D1) group were slightly less pronounced, suggesting a tendency of normalization, while in the T60 higher-dose (D2) group, the cellular alterations were markedly accentuated. The neuron degeneration seen in the present study is consistent with a previous study by Xu et al. [41], which observed rat-brain toxicity after intragastric administration of two different doses of AgNPs (1 mg and 10 mg/kg body weight). Similarly to their results, we also observed significant alterations to the integrity of the BBB, consisting of irregularly shaped capillaries with a proliferated endothelium, vacuolization, and lysis of astrocyte end-foots. Additionally, while these changes were observed in all treated groups, they were markedly accentuated in groups sacrificed 15 days post-treatment. This observation further supports not only the ability of smaller-sized nanoparticles to cross and alter the BBB but also their ability to persist and continuously inflict time-dependent damage at this level [42]. 

The main mitochondrial ultrastructural changes observed in both treated groups consisted of swelling (rarefied matrix), altered cristae (cristolysis), and elongation. Swelling and cristolysis are suggestive of mitochondrial dysfunction, while elongation has been shown to be a result of enhanced fusion activity and a typical response to stress conditions. Through fusion, mitochondria have been shown to display more efficient energy production while simultaneously being protected from degradation [43]. It is important to note that mitochondrial elongation has been described as a process that occurs during autophagy, a critical process that can act as an antioxidant mechanism, sustaining cell viability by clearing organelles and proteins damaged by excessive ROS production during oxidative stress [44]. Other ultrastructural signs of autophagy induction were observed (identification of autophagosomes) in samples from groups treated with low and high doses of AgNPs. Our results are in alignment with a previous study by Skalska et al. [45], which reported ultrastructural characteristics of autophagy induction in the rat brain after prolonged intragastric administration of a low dose of AgNPs (0.2 mg/kg body weight). 

Histological examination of samples from AgNP-treated groups revealed dose- and time-dependent pathological modifications that reflected the ultrastructural alterations observed in TEM. The primary modifications consisted of dark neurons with pericellular edema, myelin vacuolization, heterogenous neuropile, and karyorrhexis. These findings are in accordance with previously reported histological modifications induced by AgNPs [46]. 

As previous studies have highlighted that the cytotoxic properties of AgNPs primarily affect glial cells and astrocytes [47], we also performed a GFAP immunohistochemistry examination of the frontal cortex and hippocampal samples in order to assess the impact of our biosynthesized nanoparticles. While in both treatment groups, astrogliosis was observed, the astrocytes from groups sacrificed 15 days post-treatment presented a small cellular body with shortened processes. This is in stark contrast to results obtained by El-Drieny et al. [48], who, following gastric administration of gold nanoparticles (400 µg/kg rat body weight/day for 8 weeks), observed marked astrogliosis manifested through proliferation of intense GFAP-positive hypertrophic astrocytes with long processes. More recently, a study conducted by Repar et al. [49] on a human embryonic-stem-cell-derived neuron and astrocyte network found that low concentrations (0.1 μg/mL) of citrate-coated Ag nanoparticles increased the astrocyte/neuron ratio, while higher concentrations (5 μg/mL) had the opposite effect. Additionally, astrocytes exposed to Ag nanoparticle concentrations of 1 μg/mL and higher presented altered morphology with shortened processes. Within the context of the present study conditions, the marked astrogliosis and change in astrocyte morphology can be attributed to a number of occurrences, including but not limited to increased uptake of AgNPs followed by direct cellular damage, BBB and neuronal damage, and release of intercellular signaling molecules (ROS and inflammatory cytokines) [50]. 

Great importance must be given to the fact that brain tissue is particularly sensitive to the oxidative stress generated at this level by the activity of the glutaminergic system, high rate of oxygen utilization, high contents of oxidation-susceptible polyunsaturated fatty acids, high levels of transition metals that catalyze ROS generation, and relatively low concentrations of cellular antioxidants [51]. ROSs are highly reactive molecules that lead to a disruption of normal cell physiological processes by interacting with biomacromolecules (lipids, proteins, DNA). Excessive ROS production, past the capability of cellular antioxidant mechanisms, can lead to oxidative stress. One of the adverse effects of oxidative stress is the peroxidation of lipids in cellular membranes. Interestingly, under the current applied experimental conditions, a time-dependent modification in MDA levels was identified, mainly a significant increase in MDA levels in the hippocampus of rats sacrificed 15-days post-treatment and the frontal cortex of rats sacrificed immediately after treatment. This pattern could be explained by a difference in antioxidant capability and activity patterns between the two cerebral regions. The observed increase in MDA levels may indicate a time-dependent decrease in efficiency of enzymatic and non-enzymatic antioxidant systems toward removal of excessive ROS in the corresponding regions. Under physiological conditions, one of the enzymes that constitute the first line of defense against ROS build-up is CAT. Albeit less pronounced, analysis of CAT activity revealed a uniform decrease in enzyme activity across the two regions. In the hippocampus, CAT activity was lower in treated rats compared to the control group, while in the frontal cortex, a significant decrease was noted only in rats that received the higher dose of AgNPs. This observation is difficult to correlate with previous studies, as there is a discrepancy in the data relating to the influence of AgNPs on enzymatic antioxidant systems. However, similar results were obtained by Ferreira et al. [52] after prolonged intraperitoneal administration of gold nanoparticles. When analyzing the non-enzymatic glutathione system, a much clearer picture of the state of oxidative stress can be seen. The GSH/GSSG ratio was markedly decreased in the hippocampus of treated rats sacrificed immediately after treatment. This ratio then showed a tendency for normalization in treated rats sacrificed 15 days post-treatment. Although attenuated, the same modifications were observed in the frontal cortex. The increased rate of glutathione disulfide production through GSH oxidation can be attributed to ROS scavenging and the production of S-thiolated proteins [53]. GSH depletion, coupled with a decreased GSH/GSSG ratio in the rat brain, was also observed in a study by Skalska et al. [54] after a 14-day oral administration of a low dose of AgNPs (0.2 mg/kg body weight).

Depending on experimental conditions, AgNPs have been shown to display both pro- and anti-inflammatory effects. A study conducted by Gonzalez-Carter et al. [55] utilizing an in vitro microglial inflammatory model revealed that treatment with citrate-coated AgNPs was able to significantly reduce lipopolysaccharide-stimulated TNFα, ROS, and nitric oxide production in the aforementioned test model. On the other hand, Trickler et al. [56] found that AgNPs induced a size- and time-dependent pro-inflammatory response (increased levels of TNFα, IL-1B, and Prostaglandin E2) when administered to an in vitro rat BBB model. More relevant yet for the present study, CM-biosynthesized AgNPs have been shown to significantly reduce the release of NO, IL-12, and TNFα from CD68-positive macrophages in human psoriasis plaques [57]. While AgNPs have shown real potential for becoming a valuable alternative anti-inflammatory treatment, it is important to note that their impact on inflammation in a healthy in vivo model may paint a very different picture to that of inflammatory models. A previous study by Nakkala et al. [58] on a healthy rat model noted increased serum levels of TNFα and IL-6 following a 29-day oral administration of AgNPs biosynthesized with *Ficus religiosa*. Thus, in the setting of our healthy rat model treated with AgNPs-CM, we observed a time- and region-dependent change in TNFα levels. In the hippocampus of rats sacrificed immediately after treatment, a slight reduction in TNFα levels was observed, followed by a significant increase in levels solely in the rats treated with the higher dose and sacrificed 15 days post-treatment. TNFα levels in the frontal cortex of rats sacrificed immediately after treatment were considerably high, after which a tendency to normalization was noted 15 days post-treatment. Analysis of IL-6 revealed relatively uniform levels between both brain regions and groups. 

## 5. Conclusions

The aims of the present study were achieved and have demonstrated that despite their high biocompatibility, sub-chronic ingestion of a low, environmentally relevant dose of CM-biosynthesized AgNPs induced significant cellular modifications in the rat brain that continued to escalate after the exposure had ended. Dose- and time-dependent ultrastructural and histopathological modifications observed correlated with neurobehavioral changes in rats. 

## Data Availability

The data presented in this work are available upon request from the corresponding author.

## Supplementary Materials

The following supporting information can be downloaded at: https://www.mdpi.com/article/10.3390/nano12010058/s1, Figure S1: TEM images showing normal ultrastructure of the nervous tissue in the C group; Figure S2: TEM images presenting ultrastructural changes of the nervous tissue in the frontal cortex, recorded at the end of the experimental treatment; Figure S3: TEM images presenting ultrastructural changes of the nervous tissue in the hippocampus, recorded at the end of the experimental treatment; Figure S4: TEM images presenting ultrastructural changes of the nervous tissue in the frontal cortex, recorded 15 days after the end of the experimental treatment; Figure S5: TEM images presenting ultrastructural changes of the nervous tissue in the hippocampus, recorded 15 days after the end of the experimental treatment.
