# Supplementary material for: Neurobehavioral and Ultrastructural Changes Induced by Phytosynthesized Silver-Nanoparticle Toxicity in an In Vivo Rat Model
Source: Nanomaterials (Basel). 2021 Dec 26;12(1):58. doi: 10.3390/nano12010058 (PMC8746437; doi:10.3390/nano12010058)
Supplement: Supplementary file 1 [file nanomaterials-12-00058-s001.zip › nanomaterials-1474738-supplementary.pdf]

Supplementary Material

Neurobehavioral and ultrastructural changes induced by phytosynthesized Silver Nanoparticles in an *in vivo* rat model

Razvan Opris[a], Vlad Toma[b], Alina Mihaela Baciu[a], Remus Moldovan[a], Bogdan Dume[b], Alexandra Berghian-Sevastre[a], Bianca Moldovan[c], Simona Clichici[a], Luminita David[c], Gabriela Adriana Filip[a*], Adrian Florea[d]

[a] Department of Physiology, 'IuliuHatieganu' University of Medicine&Pharmacy, 1–3 Clinicilor Street, 400006, Cluj-Napoca, Romania;

[b] Department of Biochemistry&Experimental Biology, Institute of Biological Research, 48 Republicii Street, branch of NIRDBS Bucharest, 400015 Cluj-Napoca, Romania

[c] Department of Chemistry, Faculty of Chemistry & Chemical Engineering, 'Babes-Bolyai' University, 11. Arany Janos, 400028 Cluj-Napoca, Romania

[d] Department of Cell&Molecular Biology, 'Iuliu Hatieganu' University of Medicine&Pharmacy 6, Louis Pasteur Street, 400349, Cluj Napoca, Romania

Transmission electron microscopy of Capillaries and Astrocytes in the Frontal cortex and Hippocampus

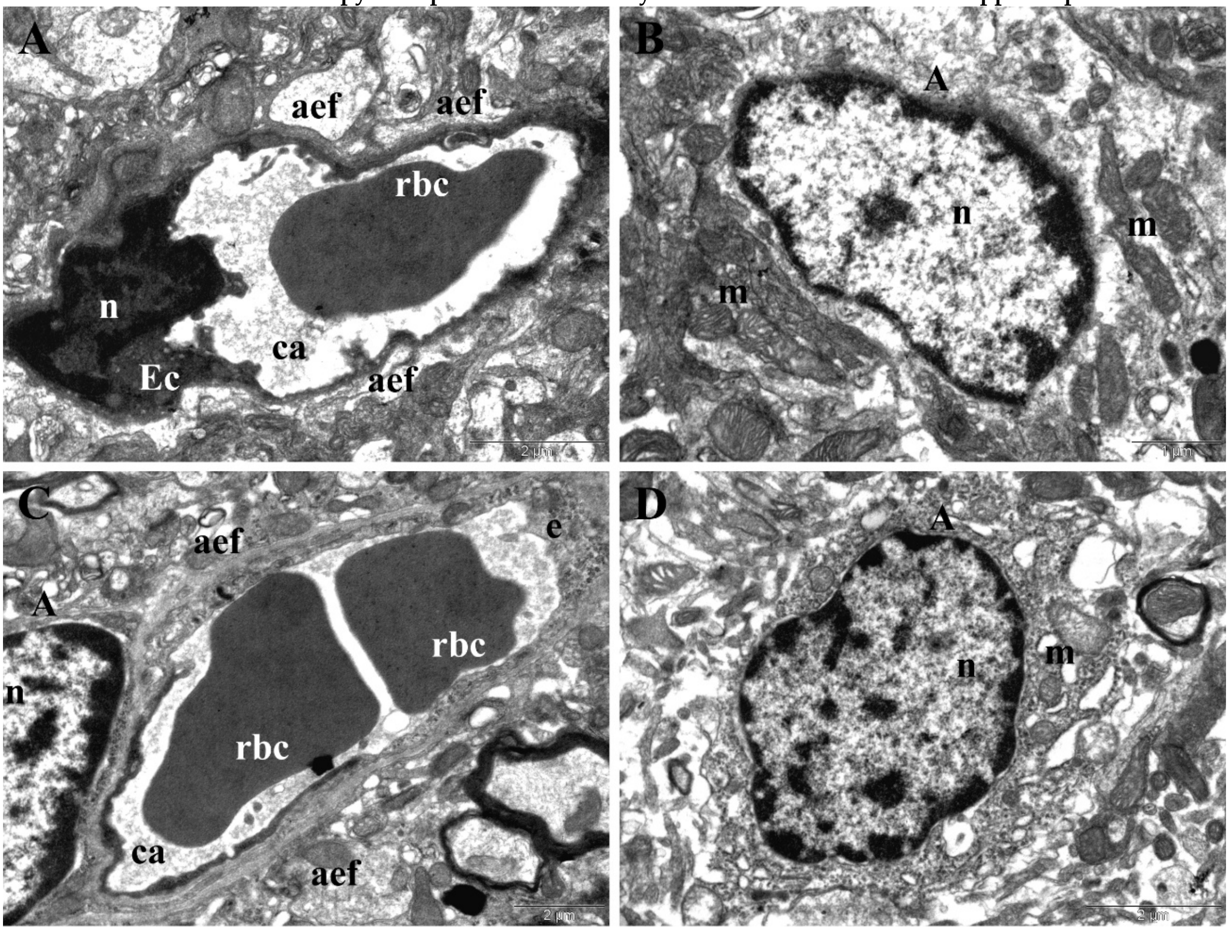

Supplementary Figure S1. TEM images showing normal ultrastructure of the nervous tissue in the C group. Frontal cortex: capillary (A) and astrocyte (B), and hippocampus: capillary (C) and astrocyte (D). A – astrocyte; aef – astrocyte end foot; ca – capillary; e – endothelium; Ec – endothelial cell; m – mitochondrion; n – nucleus; rbc – red blood cell.

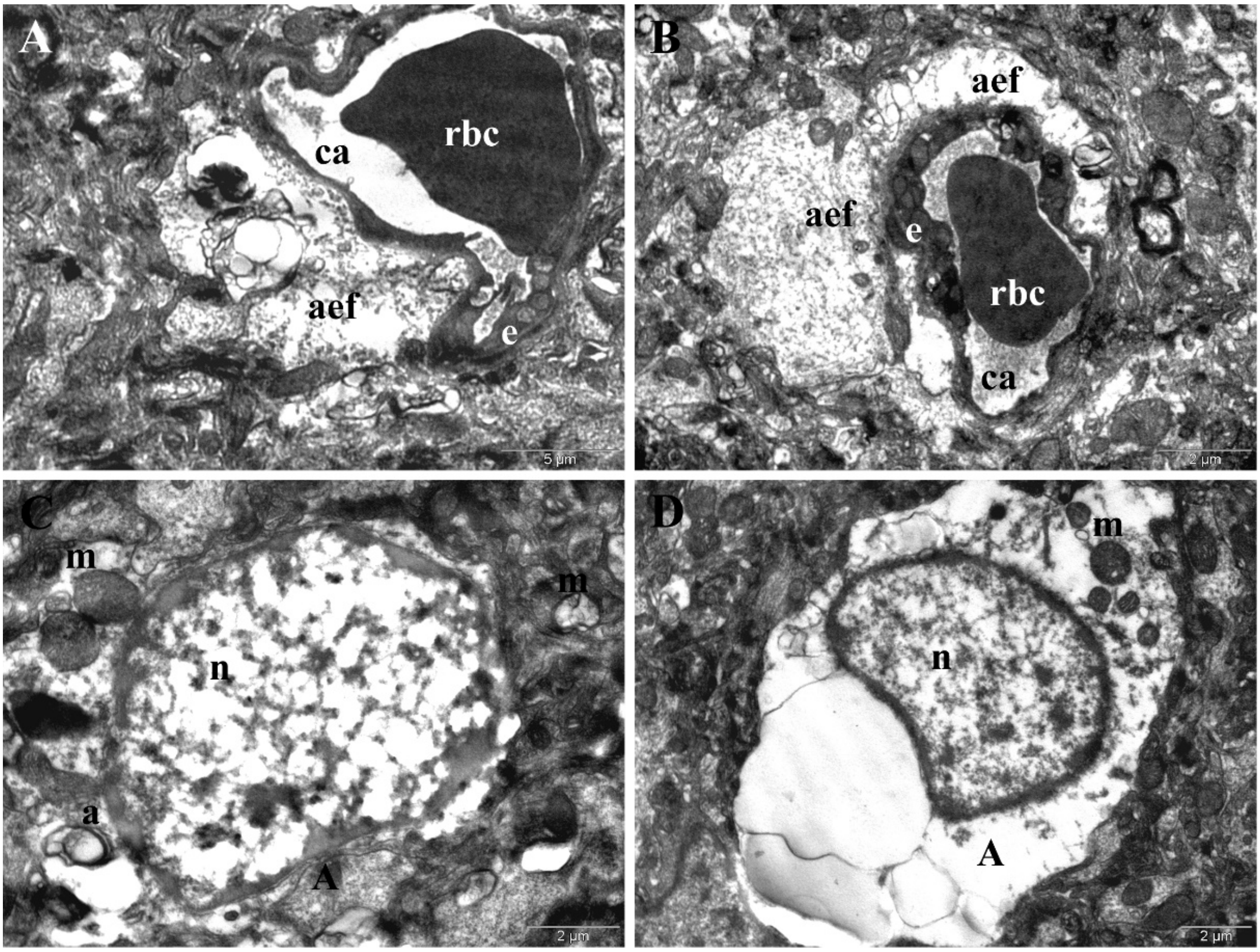

Supplementary Figure S2. TEM images presenting ultrastructural changes of the nervous tissue in the frontal cortex, recorded at the end of the experimental treatment: capillaries in T45 D1 group (A) and T45 D2 group (B), and astrocytes in T45 D1 group (C) and T45 D2 group (D), respectively. A – astrocyte; a – autophagosome; aef – astrocyte end foot; ca – capillary; e – endothelium; m – mitochondrion; n – nucleus; rbc – red blood cell.

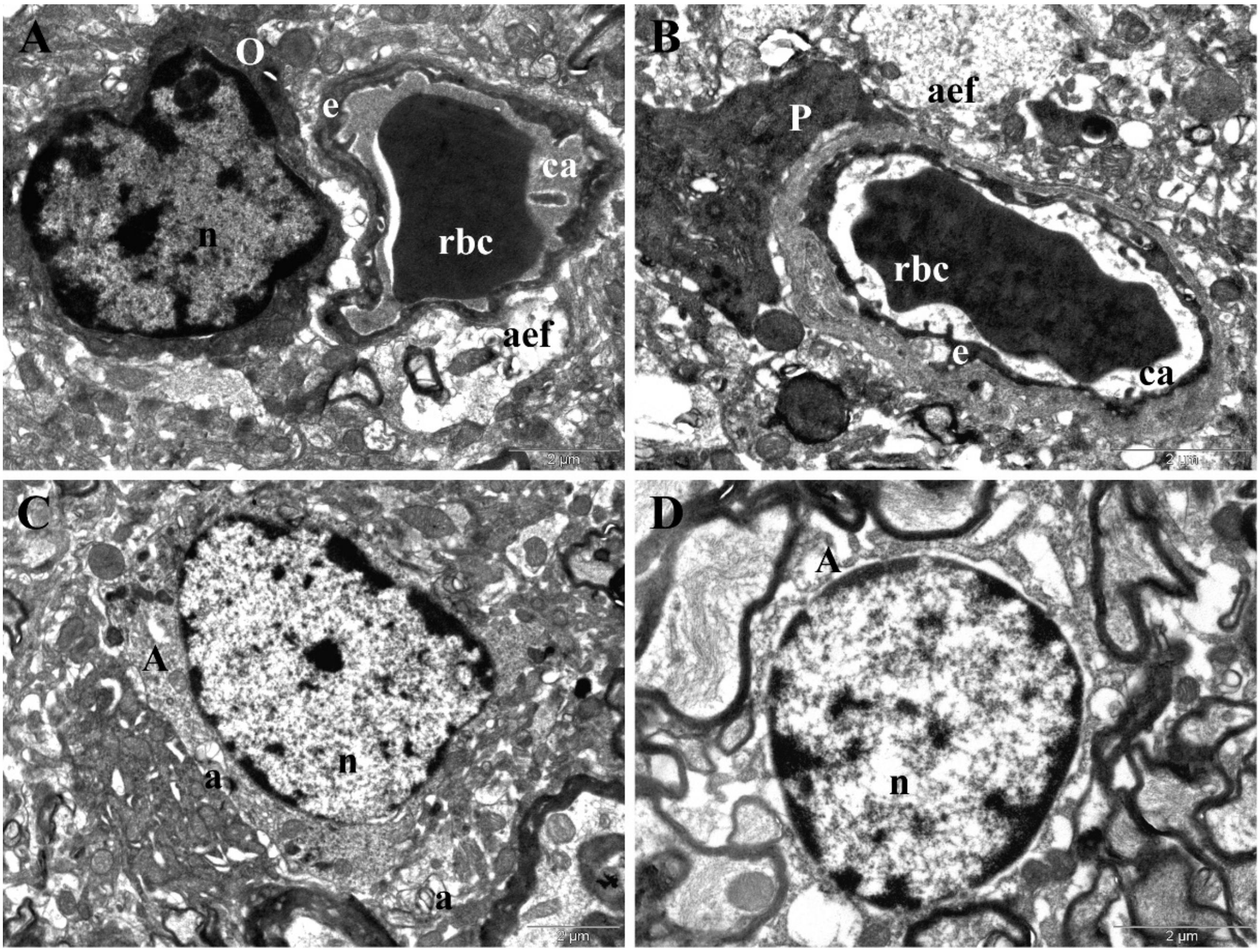

Supplementary Figure S3. TEM images presenting ultrastructural changes of the nervous tissue in the hippocampus, recorded at the end of the experimental treatment: capillaries in T45 D1 group (A) and T45 D2 group (B), and astrocytes in T45 D1 group (C) and T45 D2 group (D) respectively. A – astrocyte; a – autophagosome; aef – astrocyte end foot; ca – capillary; e – endothelium; n – nucleus; O – oligodendrocyte; P – pericyte; rbc – red blood cell.

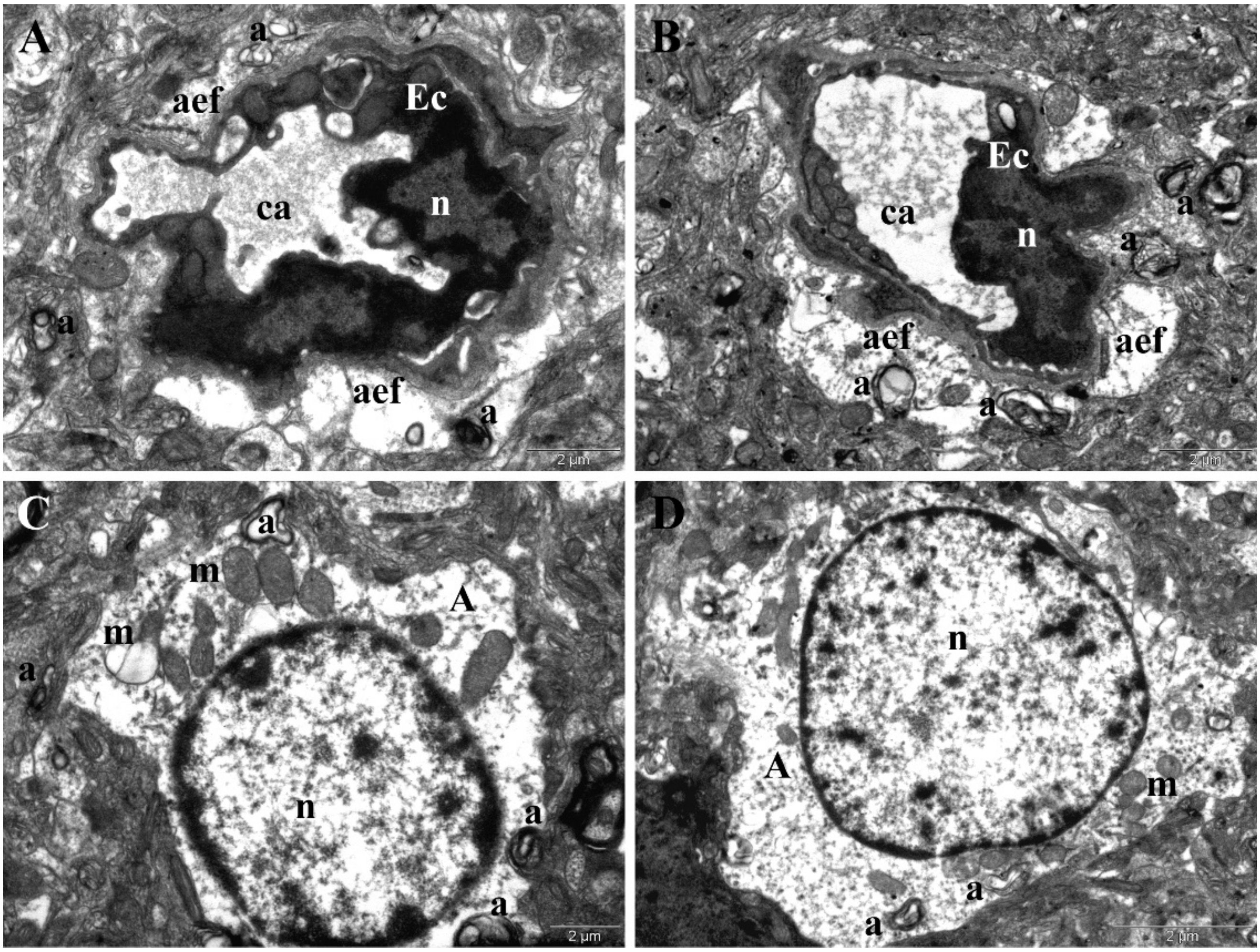

Supplementary Figure S4. TEM images presenting ultrastructural changes of the nervous tissue in the frontal cortex, recorded 15 days after the end of the experimental treatment: capillaries in T60 D1 group (A) and T60 D2 group (B), and astrocytes in T60 D1 group (C) and T60 D2 group (D) respectively. A – astrocyte; a – autophagosome; aef – astrocyte end foot; ca – capillary; e – endothelium; Ec – endothelial cell; m – mitochondrion; n – nucleus.

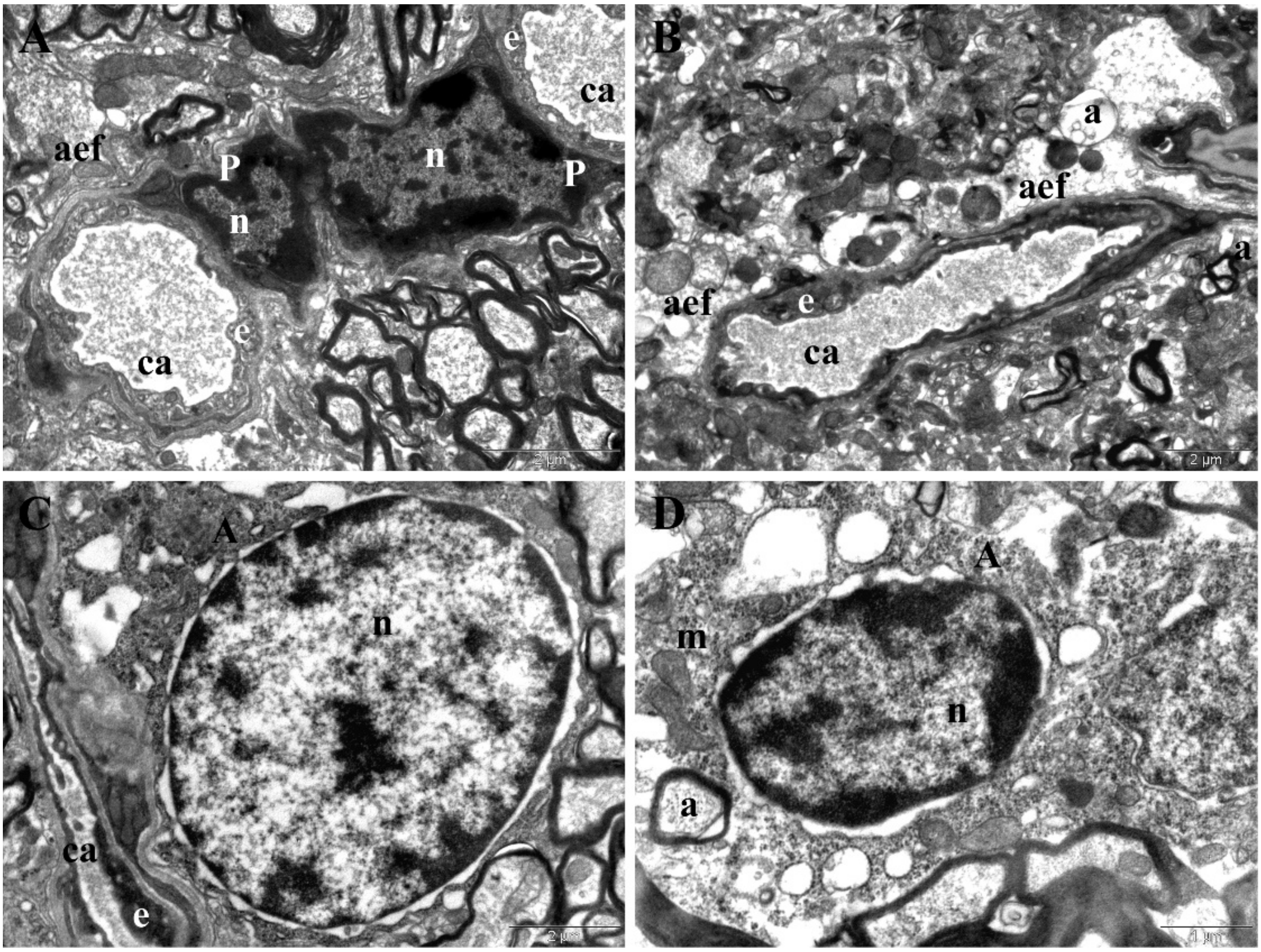

Supplementary Figure S5. TEM images presenting ultrastructural changes of the nervous tissue in the hippocampus, recorded 15 days after the end of the experimental treatment: capillaries in T60 D1 group (A) and T60 D2 group (B), and astrocytes in T60 D1 group (C) and T60 D2 group (D) respectively. A – astrocyte; a – autophagosome; aef – astrocyte end foot; ca – capillary; e – endothelium; m – mitochondrion; n – nucleus; P - pericyte.
